# Impact of an Artificial Intelligence-Guided Pulmonary Embolism Response Team (AIPERT) on Patient Transfers, Diagnosis, and Management: A Healthcare System Experience

**DOI:** 10.3390/clinpract15110207

**Published:** 2025-11-13

**Authors:** Akhil Khosla, Inderjit Singh, Jeffrey Pollak, Hamid Mojibian

**Affiliations:** 1 Department of Internal Medicine, Section of Pulmonary, Critical Care and Sleep Medicine, Yale University School of Medicine, Yale New Haven Hospital, New Haven, CT 06511, USA; inderjit.singh@yale.edu; 2 Department of Radiology and Biomedical Imaging, Section of Vascular and Interventional Radiology, Yale University School of Medicine, New Haven, CT 06511, USA; jeffrey.pollak@yale.edu (J.P.); hamid.mojibian@yale.edu (H.M.)

**Keywords:** pulmonary embolism, artificial intelligence, PE response team (PERT), triage systems, right ventricular strain, interhospital transfer, transfer coordination, computed tomography pulmonary angiography (CTPA), clinical decision support

## Abstract

Background: Pulmonary embolism (PE) is a time-sensitive condition with variable clinical presentations and outcomes. Rapid risk stratification and appropriate triage are essential for optimizing treatment and patient outcomes. Artificial intelligence (AI) offers an opportunity to enhance clinical decision-making, yet its real-world applications remain limited. Objective: The objective of this study was to describe a single healthcare system’s implementation and early experience with an AI-enabled triage tool for pulmonary embolism patients across a multi-hospital network. Methods: This retrospective observational study evaluated the deployment of an AI-based clinical decision support system within a healthcare network. The AI tool detected PE and right ventricular (RV) strain and alerted the PE response team (PERT) to facilitate timely transfer and intervention. Three cohorts were evaluated: pre-AI, Year 1 post-AI, and Year 2 post-AI. Outcomes included transfer volumes, advanced therapy rates, and hospital length of stay (LOS). Results: A total of 183 PE transfer patients were analyzed: 36 pre-AI, 72 in Year 1 post-AI, and 75 in Year 2 post-AI. Transfers increased by 100% in Year 1 (*p* = 0.0005) and 108% in Year 2 (*p* = 0.011) compared to pre-AI. Catheter-based thrombectomy increased from 10 pre-AI to 18 in Year 1 (+80%, *p* < 0.0001) and 28 in Year 2 (+180%, *p* = 0.0006). After-hours diagnosis rose from 69.4% pre-AI to 70.8% in Year 1 (*p* = 0.027) and 77.3% in Year 2 (*p* = 0.088). Surgical embolectomy showed a borderline increase in Year 2 (*p* = 0.04), though case numbers were small. Conclusions: Implementation of an AI-assisted triage platform for PE was associated with sustained increases in interhospital transfers and advanced interventions, and a reduction in hospital length of stay. These findings support the potential for AI to standardize and expedite acute PE care in a multi-hospital health system.

## 1. Introduction

Acute pulmonary embolism (PE) remains a leading cause of cardiovascular mortality globally, with incidence rates rising over the past decade [1,2]. Prompt diagnosis and timely intervention are critical, as delays are associated with increased morbidity and mortality [3,4,5]. The implementation of Pulmonary Embolism Response Teams (PERTs) has been shown to improve outcomes, including reductions in hospital length of stay and mortality [6,7,8]. However, not all hospitals have the infrastructure or staffing to support a formal PERT, and even where present, there is substantial variability in activation criteria, team composition, and treatment protocols, resulting in inconsistent care [7,9].

In most settings, acute PE management relies on the primary team to recognize the condition, seek specialty consultation, and decide whether to activate a PERT or transfer the patient to a tertiary center [8,10]. This often fragmented and reactive workflow contributes to delays in escalation of care, particularly in hospitals with limited specialty coverage or resources [9,11]. There is a growing need for scalable solutions to support early identification and streamlined triage, particularly as PE care becomes more multidisciplinary and time-sensitive [8,12].

Artificial intelligence (AI) has emerged as a promising approach, enabling earlier PE detection, faster triage, and workflow standardization [13,14]. Recent studies demonstrate that AI can detect PE on imaging with high sensitivity and specificity and can automatically calculate right ventricular (RV) to left ventricular (LV) diameter ratios to identify high-risk patients [14,15]. Automated alerts can facilitate rapid clinical action and interhospital transfers, supporting timely access to advanced therapies [16,17,18].

Since 2021, Yale New Haven Health System (YNHHS) has implemented an AI platform for system-wide PE detection, RV strain assessment, and interhospital coordination. This study describes the implementation of an AI-guided triage and transfer system and evaluates its impact on interhospital transfer volumes, advanced therapy utilization, and timeliness of diagnosis and treatment across a multi-hospital network.

## 2. Materials and Methods

### 2.1. Study Design and Setting

This was a retrospective observational study that evaluated patients with confirmed PE who were transferred within the Yale New Haven Health System (YNHHS), an academic multi-hospital health network in Connecticut. The analysis included three distinct 12-month periods: pre-AI implementation (June 2021 to May 2022), post-AI Year 1 (June 2022 to May 2023), and post-AI Year 2 (June 2023 to May 2024).

YNHHS comprises five hospitals, including Yale New Haven Hospital (YNHH), a tertiary referral center recognized as a Comprehensive PE Center of Excellence by the PERT Consortium. Advanced PE therapies, including catheter-based interventions, surgical embolectomy, and VA-ECMO, are routinely available at YNHH. The Aidoc AI platform was implemented at all hospitals within the health system.

### 2.2. AI Integration and Workflow

Aidoc (Tel Aviv, Israel) Brief Case-Triage (K232751) AI software was integrated into the radiology workflow to support PE detection and triage, Each CTPA was automatically analyzed using two algorithms. The first detected PE, while the second identified central emboli with RV strain based on an RV/LV ratio > 1.0.

**Figure 1:**
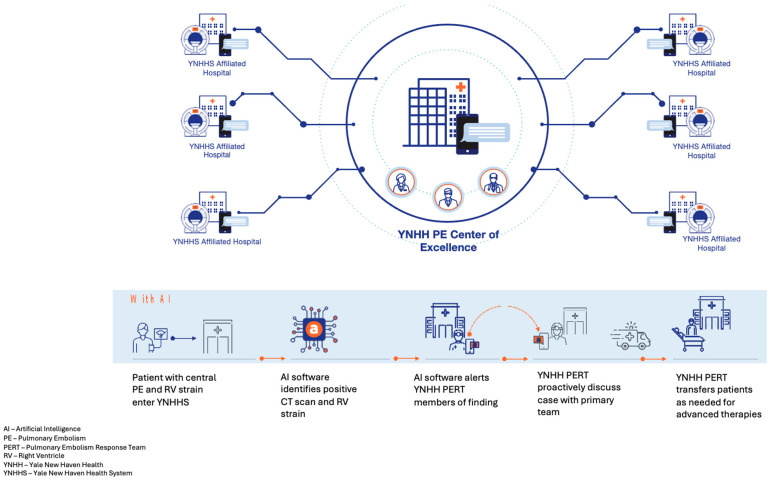
PERT Workflow with Artificial Intelligence.

If the second algorithm criterion was met, an automated alert was sent to the YNHH PERT via secure mobile notification. A designated physician reviewed the imaging, confirmed findings, and then contacted the referring hospital team to discuss patient status and determine whether transfer was warranted. A schematic overview of the AI-enabled triage and transfer workflow across the YNHHS network is shown in Figure 1.

During the study period, system expansion and protocol updates occurred. Two additional sites were integrated into the AI analysis pipeline in August 2021, followed by a third site in June 2022. These phased additions broadened the patient population eligible for transfer within the network.

### 2.3. Patient Inclusion

Patients were eligible for inclusion if they underwent CTPA during one of the study periods, had imaging-confirmed PE, and were transferred from an affiliated hospital to YNHH for PE management. We limited the study to transferred patients because only these cases were directly impacted by AI-PERT activation and centralized workflows. Patients managed exclusively at referring hospitals did not enter the standardized PERT pathway and could not be consistently captured in our institutional records.

Transfers were identified using the Epic transfer log, which records all interhospital transfers. To ensure accuracy, we cross-validated cases against admission origin fields, discharge disposition codes, and AI alert records when available. No exclusion criteria were applied based on age, sex, comorbidities, or PE location.

### 2.4. Outcomes

Primary outcomes included the volume of interhospital transfers and the number of advanced interventions performed, including thrombectomy, VA-ECMO, surgical embolectomy, and IVC filter placement. Additional process metrics included hospital length of stay (LOS), and the proportion of diagnoses occurring after hours (7:00 PM–7:00 AM). After-hours diagnoses were defined as those confirmed between 7:00 PM and 7:00 AM. Baseline patient severity was assessed using the PESI score to evaluate comparability across study groups.

### 2.5. Statistical Analysis

Descriptive statistics summarized outcomes across the three study periods. A one-sided Mann–Whitney U test was used to compare transfer rates and intervention outcomes between pre- and post-AI periods. This non-parametric test was chosen as it does not assume normal data distribution. Statistical significance was defined as *p* < 0.05. All analyses were conducted in Python (v3.12).

This study was approved for exemption by the Yale New Haven Hospital Institutional Review Board (Identifier: IRB 2000025673, Approval Date: 28 May 2019 ). Given its retrospective design and minimal risk, the IRB granted a waiver of informed consent.

## 3. Results

A total of 183 PE transfer patients were evaluated: 36 pre-AI, 72 in Year 1 post-AI, and 75 in Year 2 post-AI.

**Figure 2:**
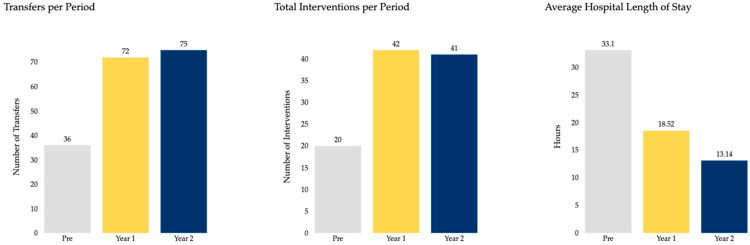
Comparison between Pre-Artificial Intelligence (AI), Year-1 and Year-2 Post AI.

**Figure 3:**
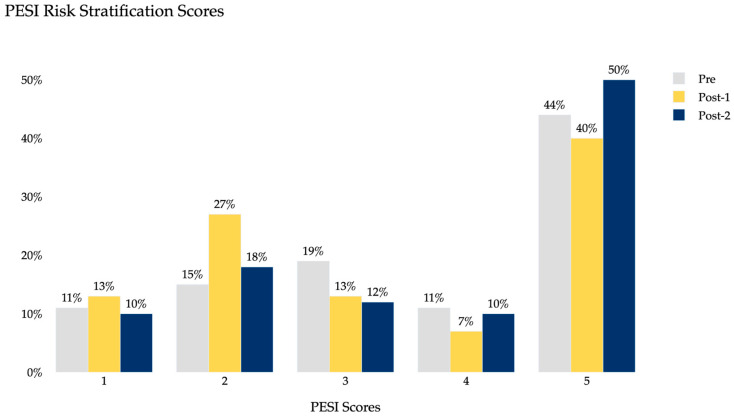
Distribution of PESI scores across pre-AI, Year 1 post-AI, and Year 2 post-AI cohorts.

Table 1 summarizes the demographic characteristics of the study population, including age, sex distribution, and BMI, which were similar across groups. Table 2 summarizes the clinical characteristics and outcome including intervention rates, after-hours diagnosis, and hospital length of stay for each period. Baseline PE severity, measured by both PESI and ESC 2019 classifications, was also comparable across pre- and post-AI periods (Figure 2 and Figure 3). PESI distributions showed the majority of patients in class 5 across all groups, while ESC classification demonstrated similar proportions of intermediate- and high-risk patients, indicating no major differences in baseline severity across cohorts.

Transfers increased by 100% in Year 1 (*p* = 0.0005) and by 108% in Year 2 (*p* = 0.011) relative to the pre-AI period. Catheter-based thrombectomy increased from 10 (pre-AI) to 18 (Year 1, +80%, *p* < 0.0001) and 28 (Year 2, +180%, *p* = 0.0006). After-hours diagnosis increased from 69.4% (25/36) pre-AI to 70.8% (51/72) in Year 1 (*p* = 0.027) and 77.3% (58/75) in Year 2 (*p* = 0.088). Hospital length of stay (LOS) decreased from 33.61 h pre-AI to 18.52 h (Year 1, −44.8%) and 13.14 h (Year 2, −60.9%). These trends are visually summarized in Figure 2, which compares transfers, total interventions, and average hospital length of stay across all three periods. No significant differences were found in VA-ECMO or IVC filter placement in either post-AI year. Surgical embolectomy showed a borderline difference in Year 2 (*p* = 0.04), though the number of cases was small.

When normalized to transfer volumes, thrombectomy was performed in 27.8% (10/36) of patients pre-AI, 25.0% (18/72) in Year 1, and 37.3% (28/75) in Year 2. After-hours diagnosis occurred in 69.4% (25/36) of patients pre-AI, 70.8% (51/72) in Year 1, and 77.3% (58/75) in Year 2. Surgical embolectomy was performed in 0% (0/36) of patients pre-AI and in Year 1, and in 2.7% (2/75) of patients in Year 2. VA-ECMO was not used pre-AI or in Year 1 and was performed in 1.3% (1/75) of patients in Year 2.

## 4. Discussion

This study showed consistent increases in transfers and interventions. Transfers doubled, and catheter-directed thrombectomy more than doubled, both reaching statistical significance. Hospital LOS decreased from about 33 h to 13 h by Year 2, reflecting more efficient workflows and faster intervention. These reductions may also improve bed availability and reduce costs in high-volume centers [6,7,12].

A major change introduced by AI was proactive transfer coordination. Previously, transfers relied on variable recognition and subjective decisions by referring teams. With real-time AI alerts for high-risk cases at the point of imaging, the YNHH PERT could immediately evaluate patients and coordinate transfers. This process doubled PE transfers and facilitated more consistent triage across the system. Importantly, the AI alerts influenced clinical decision-making by prompting earlier PERT activation at the time of imaging, thereby facilitating more timely multidisciplinary evaluation and intervention compared with pre-AI workflows.

These findings build upon prior validation studies of the Aidoc algorithm, which consistently showed strong performance in detecting PE and quantifying RV/LV ratios across diverse imaging datasets [14,15,19]. This external evidence reinforces that the observed system-level changes were grounded in an AI platform with established diagnostic reliability.

The increase in advanced interventions supports the idea that more patients received escalation of care consistent with their clinical risk. Thrombectomy rose by 80% in Year 1 and 180% in Year 2. VA-ECMO, previously unused, was applied in both post-AI years. These trends are consistent with reports showing improved outcomes among high-risk patients treated with timely advanced therapy [16,17]. However, whether greater use of advanced procedures represents an unequivocal benefit remains uncertain, since outcomes such as mortality, bleeding risk, and post-PE impairment, were not consistently available. Thus, it is possible that increased transfers and procedures exposed some patients to additional risks or costs without measurable clinical benefit.

After-hours diagnosis also improved, rising from 69% pre-AI to 77% by Year 2. Continuous AI-driven triage may reduce care delays during times of limited specialty coverage [10,18].

The increase in patient volumes across the study period likely reflects institutional changes in addition to the implementation of AI. Specifically, the Yale New Haven Health System expanded to include additional affiliated sites in 2021 and 2022, increasing the population of patients entering the standardized transfer pathway. Broader adoption of the AI-enabled workflow over time also likely contributed to the sustained rise in PE transfers observed between Year 1 and Year 2.

**Figure 4:**
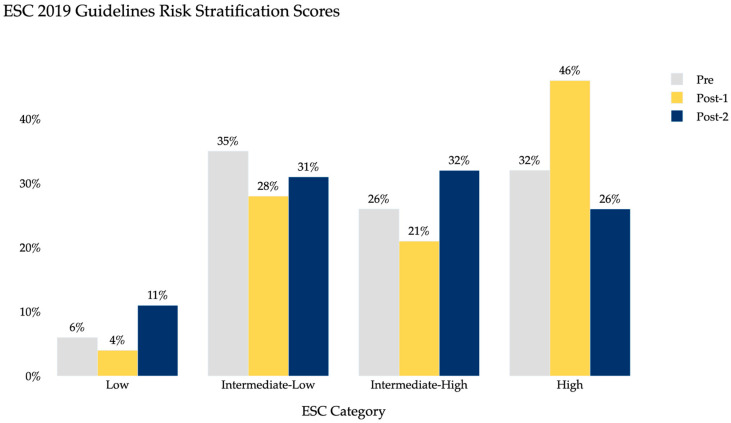
Distribution of 2019 ESC risk classifications across pre-AI, Year 1 post-AI, and Year 2 post-AI cohorts.

Baseline severity was evaluated using both PESI and 2019 ESC risk stratification, which demonstrated similar distributions across pre- and post-AI periods. Most patients were classified in higher-risk PESI categories, and the proportions of intermediate- and high-risk groups were comparable by ESC criteria (Figure 3 and Figure 4). These findings suggest that reductions in hospital LOS and increases in interventions were not primarily attributable to a shift toward less severe patients being transferred.

Despite these positive findings, this study has limitations. As a retrospective single-system analysis, results may not generalize to other settings, and unmeasured confounding is possible. Although we included PESI risk stratification to account for baseline severity, additional variables such as biomarkers and imaging markers of RV dysfunction were not uniformly available. Rare interventions (ECMO, embolectomy) limited statistical power. Importantly, patient-level outcomes such as mortality, bleeding, and functional recovery were not consistently available across hospitals in our system. The absence of these measures limits interpretation of whether increased transfers and procedures translated into improved patient health. Finally, we did not include time to diagnosis as an outcome, as our dataset combined ED presentations (typically diagnosed within hours) with inpatients who developed PE later during hospitalization (often several days after admission), resulting in values that were not clinically interpretable. We believe these derived definitions reflect real-world practice but acknowledge that they introduce complexity in interpreting absolute values. We have highlighted this as a key priority for future research.

Implementation of AI systems also carries practical barriers. Alert fatigue can reduce clinician responsiveness if notifications are too frequent or poorly targeted. Radiologist engagement is critical, as AI findings require confirmation before clinical action. Transfer logistics—particularly bed availability, transport coordination, and communication between hospitals—also affect scalability. While our system benefited from centralized workflows, broader adoption will require balancing efficiency gains with resource demands and minimizing disruptions to existing clinical practice.

Future research should include multicenter, prospective studies to validate findings, measure clinical outcomes, and assess cost-effectiveness. Evaluating barriers—such as alert fatigue, radiologist engagement, and resource allocation—will be key for scalability [11,13,19]. Additional studies should also assess if a similar triage workflow could be applicable to other areas in medicine where AI is being used in a time-sensitive manner such as stroke and aortic aneurysm.

In conclusion, AI-assisted triage and transfer across a multihospital network improved timeliness, expanded access to advanced therapies, and standardized workflows in acute PE care. These results highlight the potential for AI to extend the reach of PE Centers of Excellence and enable faster, more equitable care for high-risk vascular patients [18]. These findings demonstrate system-level changes, but further work is needed to determine whether they translate into improved patient outcomes.

## 5. Conclusions

In conclusion, our findings suggest that AI-enhanced PE triage and transfer can significantly increase interhospital transfers and expand access to advanced therapies. These results highlight the potential for AI-driven platforms to accelerate care delivery and workflow efficiency, supporting the broader mission of PE Centers of Excellence to provide timely, standardized, and equitable care. Future research should assess long-term patient outcomes and further explore the role of AI in optimizing acute PE management across diverse clinical settings.

## Figures and Tables

**Table 1:** Demographics of Patients with Pulmonary Embolism Across Pre- and Post-AI Implementation Periods.

	Pre	Post-1	Post-2
Total Patients, number (%)	36 (100)	72 (100)	75 (100)
Sex, number (%)			
Males	15 (41.67)	41 (56.94)	32 (42.67)
Females	21 (58.33)	31 (43.06)	43 (57.33)
Age Ranges, number (%)			
18–44	5 (13.89)	10 (13.89)	9 (12.00)
45–64	7 (19.44)	25 (34.72)	27 (36.00)
65–75	12 (33.33)	20 (27.78)	23 (30.67)
>75	12 (33.33)	17 (23.61)	16 (21.33)
BMI, mean (SD)	30.39 (7.98)	29.31 (8.33)	29.79 (6.80)

**Table 2:** Clinical Characteristics and Outcomes of Patients with Pulmonary Embolism Across Pre- and Post-AI Implementation Periods.

	Pre	Post-1	Post-2
OSH Transfer, number (%)	36 (100)	72 (100)	75 (100)
FlowTriever, number (%)	10 (27.78)	18 (25.00)	28 (37.33)
VA-ECMO, number (%)	0 (0)	2 (2.78)	2 (2.67)
Surgical Embolectomy, number (%)	1 (2.78)	1 (1.39)	0 (0)
IVC Filter, number (%)	9 (25.00)	21 (29.17)	11 (14.67)
After-Hours Diagnosis, number (%)	25 (69.44)	51 (70.83)	58 (77.33)
Hospital LOS (Hours), mean (SD)	33.61 (99.09)	18.52 (17.28)	13.14 (14.85)

## Data Availability

The original contributions presented in this study are included in the article. Further inquiries can be directed to the corresponding author.

## Abbreviations

The following abbreviations are used in this manuscript:

AI	artificial intelligence
LOS	length of stay
PE	pulmonary embolism
PERT	pulmonary embolism response team
RV	right ventricular
